# Homologous recombination deficiency gene panel analysis results in synchronous endometrial and ovarian cancers

**DOI:** 10.1590/1806-9282.20240534

**Published:** 2024-09-30

**Authors:** Ferah Kazanci, Zerrin Yılmaz Çelik, Mert Polat, Ferhat Karademir, Ozlem Erdem, Feride İffet Şahin, Mehmet Anil Onan

**Affiliations:** 1 Gazi University, Faculty of Medicine, Department of Gynaecologic Oncology – Ankara, Turkey.; 2 Baskent University, Faculty of Medicine, Department of Medical Genetics – Ankara, Turkey.; 3 Acıbadem University, Faculty of Medicine – İstanbul, Turkey.; 4 Gazi University, Faculty of Medicine, Department of Pathology – Ankara, Turkey.

**Keywords:** Synchronous neoplasm, Homologous recombination, Deficiency, ATM, Genes

## Abstract

**OBJECTIVE:**

The objective of this study was to analyze the genetic alterations of tumors within the scope of the homologous recombination deficiency gene panel in patients diagnosed with synchronous endometrial ovarian cancer who have been followed for over 5 years using next-generation sequencing.

**METHODS:**

DNA was isolated from the patient’s formalin-fixed, paraffin-embedded tissue blocks. Next-generation sequencing was performed using the Illumina capture-based sequencing method. Samples were sequenced using the Sophia HR Solution DNA Kit.

**RESULTS:**

Seven patients were included in this study. The ratios of likely pathogenic (LP)/pathogenic (P) somatic mutations in *ATM* (serine/threonine kinase or Ataxia-telangiectasia mutated gene), *BRCA2* (breast cancer type 2 susceptibility gene), *BARD1* (*BRCA1* associated RING domain 1), *TP53* (tumor protein p53), *BIRP1* (*BRCA1*-interacting helicase 1 gene), *PALB2* (partner and localizer of *BRCA2*), and *CHECK2* were 21 (48.8%), 8 (18.6%), 5 (11.6%), 3 (6.9%), 2 (4.6%), 2 (4.6%), and 2 (4.6%), respectively, in endometrium, and the ratios of somatic mutations in *ATM*, *BRCA2*, *TP53*, *BARD1*, *RAD54L* (DNA repair/recombination protein like), *BIRP1*, and *RAD51D* (RAD51 recombinase paralog D) were 24 (60%), 6 (15%), 5 (12.5%), 2 (5%), 2 (5%), 1 (2.5%), and 1 (2.5%), respectively, in ovary. In endometrioid-synchronous endometrial ovarian cancer cases, P/LP mutations were observed in *ATM* and *CHECK2* genes in endometrium and *ATM, BRCA2*, and *TP53* genes in ovary. In two non-endometrioid-synchronous endometrial ovarian cancer cases, *CHEK2* (checkpoint kinase 2) mutations were observed in endometrium and *ATM* and *TP53* mutations in ovary, whereas in one case, P/LP mutations in *ATM* and *TP53* genes were common in both tissues.

**CONCLUSION:**

Pathogenic variations confirming the diagnosis of synchronous endometrial ovarian cancer with genetic alterations were identified in all but one case. *ATM* gene mutation emerged as the most common alteration and has a potential association with a favorable prognosis.

## INTRODUCTION

The most common concurrent malignancies in the female genital system are endometrial (EC) and ovarian tumors (OC), observed in 10% of OC cases and 5% of ECs^1^. The histopathological criteria have been established to determine whether this condition is metastatic (MC) or synchronous endometrial ovarian cancer (SEOC)^2,3^. However, relying solely on histopathological findings to differentiate between SEOC and MC can lead to misstaging, causing potential risks by mismanagement of patients. Given the different prognostic properties of MCs and SEOCs, accurate diagnoses are crucial for determining the treatment^2,3^. Detailed histopathological features, along with molecular investigations, are recommended for the diagnosis of SEOCs.

Clonality analyses through parallel sequencing methods were conducted for the differential diagnosis of MCs and SEOCs, identifying SEOCs exhibiting the same clonality^4,5,6^. Mutations in *TP53* (tumor protein p53), *PTEN* (phosphatase and tensin homolog), *POLE* (DNA polymerase epsilon, catalytic subunit), *PIK3CA* (phosphatidylinositol-4,5-bisphosphate 3-kinase catalytic subunit alpha), *KRAS* (KRAS proto-oncogene GTPase), *ARID1A* (AT-rich interaction domain 1A), *FGFR2* (fibroblast growth factor receptor 2), and *CTNNB1* (catenin beta1) genes, as well as immunohistochemical evaluation of DNA mismatch repair (MMR) protein expression, were performed in SEOCs^4-8^. MMR deficiency was observed in 28.3% of cases with not significantly associated with survival^8^.

Homologous recombination (HR) ensures the error-free repair of deoxyribonucleic acid (DNA) double-strand breaks (DBSs) during the DNA repair process. The inability to repair DNA-DBSs effectively using the HR repair pathway is defined as HR deficiency (HRD)^9^. HRD is a relative biomarker with both predictive and prognostic values in OCs. It is estimated that approximately 41–50% of OCs and 5–15.7% of ECs exhibit HRD^10,11,12,13^.

The HRD molecular phenotype represents a positive predictive biomarker for using of poly(ADP-ribose) polymerase (PARP) inhibitors and platinum-based chemotherapy in OCs. SEOCs show a good prognosis despite containing two separate malignancies, yet there are contradictions in the adjuvant treatment approaches for these patients. The goal was to analyze the genetic alterations within the scope of the HRD gene panel in SEOC cases who have been followed for over 5 years using next-generation sequencing (NGS). Our aim is to provide appropriate target-oriented treatment planning for these patients.

## METHODS

### Study design and characterization of participants

The medical records of 1,673 patients who underwent surgery for EC and OC between July 2005 and August 2022 were reviewed. According to histopathological criteria, 43 (2.57%) SEOC and 30 (1.79%) MC cases were identified. Among the patients with histopathologically confirmed SEOCs, seven patients with an overall survival (OS) of 5 years or more were included in the study.

Clinical characteristics, menopausal status, chief complaints of patients, presence of systemic disease history, serum CA-125 level, tumor size, surgical approach, and postoperative adjuvant treatment records and pathological findings of SEOCs were evaluated. Scully criteria were used to distinguish SEOCs from MCs who presented until 2014, and after 2014, the differential diagnosis of patients was made according to the WHO criteria^2,3^. Stages of OC and EC were determined according to the FIGO 2009 classification. Each patient was followed up every 3 months for the first 2 years, every 6 months for 2–5 years, and annually thereafter. Cases operated on in this institution but not followed there, as well as cases with additional malignancies in addition to SEOC, were excluded.

### Sequencing analysis

DNA was isolated from formalin-fixed paraffin-embedded (FFPE) blocks using the Promega ReliaPrep FFPE gDNA Miniprep System kit and using Promega’s standard method recommended. NGS was performed using the Illumina capture-based sequencing method.

Samples were sequenced using the Sophia HR Solution DNA Kit. Panel genes included *ATM*, *BARD1*, *BRCA1*, *BRCA2*, *BRIP1*, *CDK12* (cyclin-dependent kinase 12), *CHEK1* (checkpoint kinase 1), *CHEK2* (checkpoint kinase 2), *FANCL* (FA complementation group L), *RAD51B* (RAD51 paralog B), *RAD51C*, *RAD51D* (RAD51 recombinase paralog D), *RAD54L* (DNA repair/recombination protein like), *PALB2* (partner and localizer of *BRCA2*), *PPP2R2A*, and *TP53*. Data analysis was performed using the Sophia DDM platform and in silico tools (https://franklin.genoox.com/clinical-db/; https://www.ncbi.nlm.nih.gov/clinvar/), and variants were classified according to the American College of Medical Genetics criteria. In our study, alterations with a variant allele frequency rate of 5% or higher in tumor tissue were evaluated.

### Statistical analysis

The Statistical Package for Social Sciences, version 22.0 (SPSS Inc., Chicago, USA), a computer software package, was used for the statistical analysis of research data. For categorical variables, the number and percentage were provided, while for continuous variables, the mean±standard deviation and median values were presented.

### Ethical approval

Ethical approval for the project with the protocol number KA21/502 was obtained from the Institutional Review Board of Başkent University Medical and Health Sciences Research Committee on January 13, 2022.

## RESULTS

The average age of patients was 49 years, and their demographic and clinical characteristics are presented in Table 1. Four of them had endometrioid-type histopathology in both ovary and endometrium (EN-SEOC), while three showed non-endometrioid-type (NE-SEOC) (Table 2).

**Table 1:** Demographic, clinical, and histopathological findings of the patients.

	n (%)	Grade (n, %)	Evre (n, %)
Age (med/min–max)	49 (38–64)		
Nullipar/mıltipar	3 (42.9)/4 (57.2)		
BMI (med/min–max)	30 (27–32)		
Symptoms at the time of presentation			
Abnormal uterine bleeding	2 (28.6)		
Pelvic pain/abdominal distention	3 (42.9)/2 (28.6)		
History of other disease (-)	4 (57.1)		
DM/HT/DM+HT	2 (14.3)/1 (14.3)		
Ca-125 levels (med/min–max)	16 (11–776)		
Preop.Biopsy (+)/(-)	2/5 (28.6/71.4)		
TAH+BSO+PPLND+Omentektomi	0/7 (100)		
Malign cytology (+)/(-)	1/6 (14.3/85.7)		
Histology of endometrium			
Endometriod	6 (85.7)	G1-2 (28.6)	Ia-6 (85.7)
Nonendometriod	1 (14.3)	G2-4 (57.1)	IIIc1-1 (14.3)
Serous	1 (14.3)	G3-1 (14.3)	
Malign invasion (Invasion depth/<1/2)	2 (28.6)/5 (71.4)		
Cervical invasion (+)/(-)	1/6 (14.3/85.7)		
Endometrial LVSI (+)/(-)	1/6 (14.3/85.7)		
Ovarian cytology			
Endometrioid	4 (57.1)	G1-1 (14.3)	Ia-4 (57.1)
Nonendometrioid	3 (42.9)	G2-3 (42.9)	Ib-1 (14.3)
Serous	3 (42.9)	G3-3 (42.9)	IIIc-2 (28.6)
Ovarian LVSI (+)/(-)	2/5 (28.6/71.4)		
Endometriosis (+)/(-)	4/3 (57.1/42.9)		
RT+CT/CT	4 (57.1)/3 (42.9)		
OS (med/min–max)	101 (61–128)/		
RFS (med/min–max)	101 (13–128)		
Total (patient, n)	7		

**Table 2:** Clinical and molecular findings of patients.

	AN	AE	GK	SG	MA	DU	SÇ
	EN-SEOC				NE-SEOC		
Age	47	49	49	38	64	57	64
Ca-125 (IU/mL)	16	12	11	90	776	14	361
Endometrium histology	End.	End.	End.	End.	End	Ser.	End.
Endometrium grade	2	2	2	2	1	3	1
Myometrial invasion	<1/2	<1/2	Superficial	<1/2	<1/2	<1/2	Superficial
Cervical stromal invasion	(-)	(-)	(-)	(-)	(-)	(+)	(-)
Endometrial hyperplasia/polyp	(-)/(-)	(-)/(-)	(-)/(+)	(-)/(-)	(-)/(-)	(-)/(-)	(-)/(+)
LVSI	(-)	(-)	(-)	(-)	(-)	(+)	(-)
Tumor diameter (cm)	4	7	2.5	6	0.4	4	1
Endometrial mutation	-	-	*ATM* ^1^	*CHEK2*	*CHEK2*	*ATM* ^2^ *TP53* ^2^ *BRCA2*	*-*
Ovarian histology	End.	End.	End.	End.	Ser.	Ser.	Ser.
Ovarian grade	2	1	2	2	3	3	3
Ovarian endometriosis	(+)	(+)	(+)	(+)	(-)	(-)	(-)
LVSI	(-)	(-)	(-)	(-)	(+)	(+)	(-)
Tumor diameter (cm)	4	30	5	8	9	4	3
Ovarian mutation	*ATM*	*ATM* *BRCA2* *TP53*	*ATM* ^1^	*ATM*	*ATM* *TP53*	*ATM* ^2^ *TP53* ^2^	*TP53*
Adjuvant therapy	RT+KT	RT+KT	KT	RT+KT	RT+KT	KT	KT
Recurrence	-	-	-	-	Liver	-	-
Mortality	-	-	-	-	-	-	-
OS	65	128	114	121	65	101	70
RFS	65	128	114	121	13	101	70

^1^Different mutations in the same gene in both endometrium and ovary;

^2^identical mutations in the same gene in both endometrium and ovary. EN-SEOC: endometrioid synchronous endometrial ovarian cancer; NE-SEOC: nonendometrioid synchronous endometrial ovarian cancer; End: endometrioid; Ser: serous; RT: radiotherapy; KT: chemotherapy; OS: overall survival; RFS: recurrence-free survival; LVSI: lymphovascular space invasion.

According to the sequencing results, the frequencies of likely pathogenic (LP)/pathogenic (P) somatic mutations (SMs) in *ATM*, *BRCA2*, *BARD1*, *TP53*, *BIRP1*, *PALB2*, and *CHECK2* genes were 21 (48.8%), 8 (18.6%), 5 (11.6%), 3 (6.9%), 2 (4.6%), 2 (4.6%), and 2 (4.6%), respectively, in endometrium and the ratios of LP/P SMs in *ATM*, *BRCA2*, *TP53*, *BARD1*, *RAD54L*, *BIRP1*, and *RAD51D* were 24 (60%), 6 (15%), 5 (12.5%), 2 (5%), 2 (5%), 1 (2.5%), and 1 (2.5%), respectively, in ovary (Table 3).

**Table 3:** Distribution of P/LP mutations observed in the endometrium and ovarian tissues in synchronous endometrial ovarian cancer cases.

	Gene	cDNA	Protein	Reference sequence	Variant detection rate	ACMG criteria	E	O
*AE*	*ATM*	c.818_819insC	p.(Ser274Phefs*8)	NM_000051	5.8	PVS1, PM2	(-)	(+)
	*BRCA2*	c.2141_2143delinsCAT	p.(Glu714_Gly715delinsAla*)	NM_000059	7.2	PVS1, PM2	(-)	(+)
	*TP53*	c.707A>G	p.(Tyr236Cys)	NM_000546	10.8	PM2,PM1,PP3,PM5 PP2, PP5	(-)	(+)
*GK*	*ATM*	c.818_819insC	p.(Ser274Phefs*8)	NM_000051	7	PVS1, PM2	(+)	(-)
		c.1236-2A>T	p.(?)	NM_000051		PVS1, PP5, PM2	(-)	(+)
*AN*	*ATM*	c.818_819insC	p.(Ser274Phefs*8)	NM_000051	5.3	PVS1, PM2	(-)	(+)
		c.832del	p.(Val278Serfs*42)	NM_000051	5	PVS1, PM2	(-)	(+)
*SG*	*ATM*	c.818_819insC	p.(Ser274Phefs*8)	NM_000051	6.2	PVS1, PM2	(-)	(+)
		c.832del	p.(Val278Serfs*42)	NM_000051	5.6	PVS1, PM2	(-)	(+)
	*CHEK2*	c.1549C>T	p.(Arg517Cys)	NM_001005735	6.5	PM2,PM5,PM1, PP3	(+)	(-)
*SÇ*	*TP53*	c.859G>T	p.(Glu287*)	NM_000546	10.1	PVS1, PM2	(-)	(+)
		c.532del	p.(His178Thrfs*69)	NM_000546	7.6	PVS1, PM2, PP5	(-)	(+)
*MA*	*ATM*	c.818_819insC	p.(Ser274Phefs*8)	NM_000051	5.2	PVS1, PM2	(-)	(+)
	*CHEK2*	c.721+1G>A	p.(?)	NM_001005735	5.4	PVS1, PM2, PP5	(+)	(-)
	*TP53*	c.641A>G	p.(His214Arg)	NM_000546	59.8	PS3,PM2,PM1, PP3, PM5, PP2, PP5, PS4	(-)	(+)
*DU*	*ATM*	c.818_819insC	p.(Ser274Phefs*8)	NM_000051	6.4/8.3	PVS1, PM2	(+)	(+)
		c.832del	p.(Val278Serfs*42)	NM_000051	5.2/7	PVS1, PM2	(+)	(+)
		c.2141_2143delinsCAT	p.(Glu714_Gly715delinsAla*)	NM_000059	5.3	PVS1, PM2	(+)	(-)
		c.859G>T	p.(Glu287*)	NM_000546	69.6/22.4	PVS1, PM2	(+)	(+)

E: endometrium; O: ovarian.

In EN-SEOC cases, P/ LP mutations were observed in the *ATM* and *CHECK2* genes in endometrium and in the *ATM*, *BRCA2*, and *TP53* genes in ovary (Table 2). In addition to histopathological findings, different mutations in the genes identified through the analysis of tissues in all EN-SEOCs also supported the notion that they originated from different primers (Table 2). In NE-SEOC, in two cases with different histologies, *CHEK2* mutations in endometrium and *ATM* and *TP53* mutations in ovary supported the finding of two separate primary tumors with additional genetic changes besides histopathological criteria (Table 2). In the case showing the same serous histology in both ovary and endometrium in NE-SEOC, there were common P/ LP mutations in the *ATM* and *TP53* genes. Despite being serous histology and grade 3, this patient showed an unexpectedly good prognosis (OS 101 months), although considered MC.

## DISCUSSION

Women diagnosed with SEOC have a better prognosis compared to MCs. SEOC cases are evaluated as stage Ia, while in the MC group, they are considered as stage IIIa concerning EC or stage II concerning OC. Adjuvant therapy is not needed in stage Ia cases, while it is required in stage II and IIIa diseases^2^. In this study, the HRD gene panel was examined in seven SEOC cases and having survival rates exceeding 5 years. P/LP variations were identified, confirming SEOC diagnosis with genetic changes, except for one case.

In the analysis of single-gene mutations in SEOCs, the absence of common genetic changes indicates independent pathogenesis in each region, signifying they are SEOCs^14^. However, as the analyses used to focus on a limited part of the genome, common alterations may not have been detected in at least some of the ECs and OCs. Recent genomic studies have described clonal relationships among endometrioid-type SEOCs^4,5,6^. Anglesio et al. explained that the presence of identical genetic characteristics in primary SEOC cases in both endometrium and ovary, based on histopathological criteria, is confined to areas that are physically accessible and compatible with the microenvironment, showing limited MC potential^5^.

Chao et al., examining 14 cases of EN-SEOC and two cases of NE-SEOC using massive parallel sequencing, found common SMs in 13 of the 14 EN-SEOC cases, contrary to histopathological criteria. In two NE-SEOC cases, they detected different SMs. In contrast to Chao’s findings, this study revealed distinct SMs in all four EN-SEOCs and in two out of three NE-SEOCs^15^.

Studies claiming that SEOCs sharing the same endometrioid histology generally result from MC spread from one organ to another due to their carrying the same clonality feature^4,5,16^, alongside studies demonstrating molecular genetic changes occurring at different frequencies, suggesting different processes play a role in tumor formation or progression^17^. In research directly comparing the mutation profiles of cases with endometrioid carcinoma of the ovary and endometrium, loss of expression in *ARID1A*, *PTEN*, and *MSI-H* is more commonly observed in the endometrium than in the ovary, while *CTNNB1* mutation is statistically more prevalent in the ovary than in the endometrium^17^. In our study, we detected a higher frequency of mutations in the *CHEK2* gene in the endometrium and in the *ATM* gene in the ovary in endometrioid-type tumors.

Various parameters determining the prognosis in SEOC cases have been identified using clinical and histopathological features^1^. Although recent genomic studies have revealed that SEOC cases with different histopathologies are not independent primary tumors due to their clonal relationships, patients with these tumors often paradoxically exhibit good clinical outcomes. Researchers interpreted the paradoxically good prognosis of patients with clonally related SEOC as a result of “precursor escape.” In this hypothesis, precursor cells of EC spread beyond the uterus to reach the pelvis and eventually develop into OC under an increasing mutation burden^18,19^. This process would require initial genetic damage, leading to an intraepithelial lesion in the fallopian tube; cells from this lesion could escape and later manifest as advanced OC without an obvious tubal carcinoma in the pelvis. In our study, we explained the good prognosis observed in NE-SEOC cases with common mutations, despite unfavorable prognostic factors such as serous histology and grade 3 tumors, through the “precursor escape” hypothesis.

The presence of microsatellite instability and *POLE* mutations has been reported to be associated with a favorable prognosis in cases of SEOC^8,14^. *ATM* gene mutation has been identified as an independent favorable prognostic criterion for ECs^20^. It has been found that ATM kinase prevents malignancy in the endometrium through the stimulation of progesterone. T lymphocytes, especially cytotoxic lymphocytes, are often found in higher quantities in tumors with *ATM* mutations that infiltrate the tumor^20^. Therefore, *ATM* mutations have been suggested as an independent prognostic factor and a potential biomarker for immune checkpoint therapy in EC^20^. In our study, mutations in the *ATM* gene were more frequently detected than in other genes in the HRD panel, and all these patients had OS rates of more than 5 years. Although the number of patients in our study is limited, our findings are qualitative and supportive. Ishikawa et al. determined the frequency of SMs in *TP 53* in SEOCs, reporting rates of 37.5% in endometrium and ovary^14^. Furthermore, unlike Ishikawa, we observed a lower frequency of TP53 mutations in both endometrium (6.9%) and ovary (12.5%). Pathogenic mutations in the TP53 gene are associated with poor prognosis^6^, and the lower frequency of mutations in this gene in our patients was considered to contribute to their good prognosis.

Patients with mutations in *BRCA1/2* and other HR-DNA repair genes have achieved successful survival rates with targeted therapies using PARP inhibitors^11^. Mutations in HR genes are not limited to serous histology but are also present in non-serous histologies^12^. In our study, although EN-SEOCs in four cases, P/LP mutations were detected in *ATM* and *CHECK2* genes in endometrium and in *ATM*, *BRCA2*, and *TP53* genes in ovary, supporting this finding.

Upon retrospective evaluation of our study, the average OS of cases was 101 months, with four patients receiving both radiotherapy and chemotherapy and three patients receiving only chemotherapy. They were exposed to the adverse effects of these adjuvant treatments.

## CONCLUSION

This study examined SMs in HRD panel genes among SEOC cases to identify patients suitable for targeted therapy. The *ATM* gene mutation emerged as the most prevalent alteration, with a potential link to a favorable prognosis. Nevertheless, given the limited case count, further validation through larger case series publications is warranted to confirm the accuracy of this observation.
